# Production of subtilisin proteases in bacteria and yeast

**DOI:** 10.18699/VJ21.015

**Published:** 2021-02

**Authors:** A.S. Rozanov, S.V. Shekhovtsov, N.V. Bogacheva, E.G. Pershina, A.V. Ryapolova, D.S. Bytyak, S.E. Peltek

**Affiliations:** Kurchatov Genomic Center of the Institute of Cytology and Genetics of Siberian Branch of the Russian Academy of Sciences, Novosibirsk, Russia Institute of Cytology and Genetics of Siberian Branch of the Russian Academy of Sciences, Laboratory of Molecular Biotechnologies, Novosibirsk, Russia; Kurchatov Genomic Center of the Institute of Cytology and Genetics of Siberian Branch of the Russian Academy of Sciences, Novosibirsk, Russia Institute of Cytology and Genetics of Siberian Branch of the Russian Academy of Sciences, Laboratory of Molecular Biotechnologies, Novosibirsk, Russia; Kurchatov Genomic Center of the Institute of Cytology and Genetics of Siberian Branch of the Russian Academy of Sciences, Novosibirsk, Russia Institute of Cytology and Genetics of Siberian Branch of the Russian Academy of Sciences, Laboratory of Molecular Biotechnologies, Novosibirsk, Russia; Kurchatov Genomic Center of the Institute of Cytology and Genetics of Siberian Branch of the Russian Academy of Sciences, Novosibirsk, Russia Institute of Cytology and Genetics of Siberian Branch of the Russian Academy of Sciences, Laboratory of Molecular Biotechnologies, Novosibirsk, Russia; Innovation Centre “Biruch-NT”, Malobykovo village, Belgorod region, Russia; Innovation Centre “Biruch-NT”, Malobykovo village, Belgorod region, Russia; Kurchatov Genomic Center of the Institute of Cytology and Genetics of Siberian Branch of the Russian Academy of Sciences, Novosibirsk, Russia Institute of Cytology and Genetics of Siberian Branch of the Russian Academy of Sciences, Laboratory of Molecular Biotechnologies, Novosibirsk, Russia

**Keywords:** subtilisin, subtilase, protease, alkaline serine protease, Pichia pastoris, Bacillus subtilis, biotechnology, genetic engineering, cultivation, субтилизин, субтилаза, протеаза, щелочная сериновая протеаза, Pichia pastoris, Bacillus subtilis, биотехнология, генетическая инженерия, культивирование

## Abstract

In this review, we discuss the progress in the study and modification of subtilisin proteases. Despite longstanding applications of microbial proteases and a large number of research papers, the search for new protease genes, the construction of producer strains, and the development of methods for their practical application are still relevant and important, judging by the number of citations of the research articles on proteases and their microbial producers. This enzyme class represents the largest share of the industrial production of proteins worldwide. This situation can explain the high level of interest in these enzymes and points to the high importance of designing domestic technologies for their manufacture. The review covers subtilisin classification, the history of their discovery, and subsequent research on the optimization of their properties. An overview of the classes of subtilisin proteases and related enzymes is provided too. There is a discussion about the problems with the search for (and selection of) subtilases from natural strains of various microorganisms, approaches to (and specifics of) their modification, as well as the relevant genetic engineering techniques. Details are provided on the methods for expression optimization of industrial subtilases of various strains: the details of the most important parameters of cultivation, i.e., composition of the media, culture duration, and the influence of temperature and pH. Also presented are the results of the latest studies on cultivation techniques: submerged and solid-state fermentation. From the literature data reviewed, we can conclude that native enzymes (i.e., those obtained from natural sources) currently hardly have any practical applications because of the decisive advantages of the enzymes modified by genetic engineering and having better properties: e.g., thermal stability, general resistance to detergents and specific resistance to various oxidants, high activity in various temperature ranges, independence from metal ions, and stability in the absence of calcium. The vast majority of subtilisin proteases are expressed in producer strains belonging to different species of the genus Bacillus. Meanwhile, there is an effort to adapt the expression of these enzymes to other microbes, in particular species of the yeast Pichia pastoris.

## Abstract (ru)

В настоящей работе мы рассматриваем прогресс в изучении и модификации субтилизиновых протеаз. Несмотря на длительное время применения микробиальных протеаз и значительное число работ, посвященных их исследованию, поиск новых генов протеаз, создание продуцентов и развитие методов их применения остаются актуальными, о чем говорит высокий уровень цитирования публикаций, описывающих протеазы и их продуценты. На данный класс ферментов приходится максимальный объем производства промышленных белков в мире, что объясняет большой интерес к нему. Это говорит о чрезвычайно высокой важности получения собственных технологий их производства. В статье представлены сведения о классификации субтилизинов, истории их открытия и дальнейших работ по оптимизации их свойств. Дан обзор классов субтилизиновых протеаз и родственных им ферментов. Проанализированы проблемы поиска и отбора субтилаз из природных штаммов различных микроорганизмов, пути и особенности их модификации и используемые при этом методы генетической инженерии. Детально изучены методы оптимизации продукции промышленных субтилаз у различных штаммов, касающихся важнейших аспектов культивирования: состава среды, времени культивирования, влияния температуры и pH. Приводятся результаты последних исследований по техникам культивирования – глубинному и твердофазному культивированию. На основании рассмотренных литературных данных можно заключить, что в настоящее время практически не применяются нативные, т.е. обнаруженные в природе ферменты, в связи с решающими преимуществами, предоставляемыми белками, модифицированными при помощи генной инженерии и обладающими улучшенными свойствами: термостабильностью, общей устойчивостью к детергентам и специфической – к различным окислителям, высокой активностью в разных диапазонах температур, независимостью от ионов, стабильностью в отсутствие кальция и т.д. Большинство субтилизиновых протеаз синтезируется в штаммах-продуцентах, относящихся к разным видам рода Bacillus. В то же время ведутся работы по адаптации синтеза этих ферментов в других микроорганизмах, в частности дрожжей Pichia pastoris.

## Introduction

Proteases are enzymes that degrade proteins via the hydrolysis of peptide bonds. Proteases correspond to the general enzyme class designated as EC 3.4.X.X (Garcia-Carreno, Del Toro, 1997). Endopeptidases act most strongly on intact proteins; they cleave peptide bonds of nonterminal amino acid residues. Exopeptidases sever peptide bonds between amino acid residues at the end of a polypeptide chain. They are categorized into amino- and carboxy-peptidases depending on which end (N- or C-terminus) they remove amino acids from (Barrett, McDonald, 1986). Proteases are subdivided into families in accordance with their mechanism of action. According to database MEROPS (http://merops.sanger.ac.uk) (Rawlings et al., 2014), the following protease families are known: asparagine, cysteine, glutamine, serine and threonine peptidases, metalloproteinases, mixed peptidases, and peptidases with an unknown mechanism of action

Peptidases are present in all life forms. Today, the most popular proteases are those from prokaryotes, mainly bacteria, because of their excellent potential for various technological applications. Given that proteases are needed in large amounts, the cost of production is as important as protease characteristics; as a consequence, in most cases, proteases are manufactured by means of bacteria. Microorganisms can produce proteases faster and more cheaply than mammalian and plant cells can; the enzyme manufacture is not affected by the climate or changes of seasons or by regulatory or ethical problems. Besides, extracellular enzymes expressed by microorganisms are usually preferred because subsequent processing is simpler, meaning even lower costs (Tufvesson et al., 2010). In terms of a combination of characteristics (activity, pH and temperature ranges, and production costs), subtilisins or subtilases have turned out to be the most popular class of proteases.

Subtilases are one of the largest classes of serine proteases that are encoded in the genomes of all life forms including viruses. By amino acid sequence, subtilases are subdivided into six families: subtilisins, thermitases, proteinases K, lantibiotic peptidases, kexins, and pyrolisins. Subtilisins in turn are categorized into several subfamilies: true subtilisins, highly alkaline proteases, intracellular proteases, intermediate subtilisins, and high-molecular-weight subtilisins.

All the subfamilies of subtilisins hold promise for biotechnology. The first alkaline serine protease that gained widespread use was subtilisin A (EC 3.4.21.62), which is an alkaline serine protease from Bacillus subtilis. The enzyme owes its name to the species of its bacterial producer (Ottesen, Svendsen, 1970; Ikemura et al., 1987). The history of discovery and study of subtilisins started at a research center of a beer-brewing company called Carlsberg, and the first enzyme to be described is named “subtilisin Carlsberg” (Smith et al., 1966).

The catalytic center of serine proteases is formed by three amino acid residues: Asp-32, His-64, and Ser-221. Because the amino acid residue carrying out the nucleophilic attack is Ser-221, subtilisins and the related proteolytic enzymes are called serine proteinases. Among the highly alkaline proteases, there is an enzyme isolated from strain Bacillus sp. KSM-K16 (Kobayashi et al., 1995). Its optimum of activity is at 55 °C and pH 12.3. This enzyme is employed in the industry in complex with a detergent, as is the case for related highly alkaline proteases, Savinase and Maxacal. Intermediate subtilisins are somewhere between true subtilisins and highly alkaline proteases and include some promising enzymes. For instance, the ALTP enzyme isolated from Alkaliphilus transvaalensis (Kobayashi et al., 2007) shows maximal activity at very high temperatures and pH, namely, at 70 °C and pH>12.6. Nonetheless, ALTP can also perform a catalytic function at lower temperatures and pH. The phylogenetic tree based on the amino acid sequences of subtilisin proteases is presented in the Figure.

**Fig. 1.**
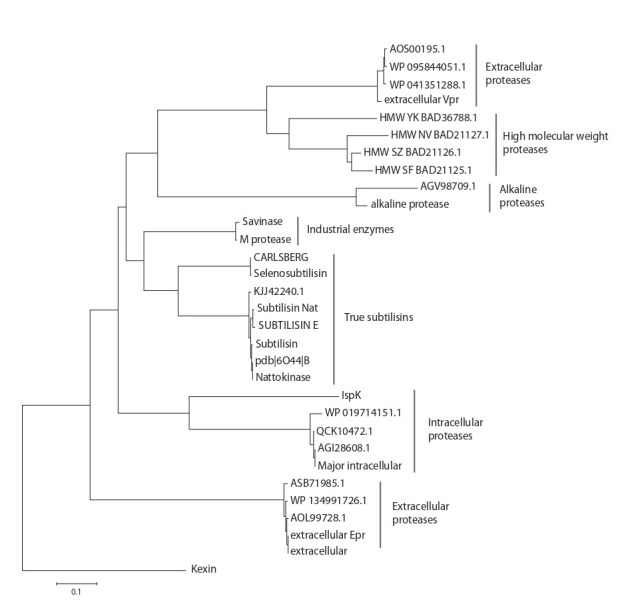
Subtilisin proteases and the related enzymes of B. subtilis as well as some industrial enzymes.

Intracellular proteases are rather poorly studied in comparison with the above subfamilies. The reason is that they are active at lower pH, which is characteristic of the cytoplasm. For example, the intracellular protease from B. megaterium (Jeong et al., 2018) at 50 °C shows an optimum of activity at pH 6.0–7.0.

From alkalophilic Bacillus spp., researchers isolated a set of high-molecular-weight subtilisins (Okuda et al., 2004) ~650 amino acid residues long (size of the precursor: 800 amino acid residues). Their optimal pH is 10.5–11.0, and optimal temperatures for activity are 40–45 °C.

Bacteria are most widely used as a microbial producer of proteases, and the genus Bacillus is the most famous source among them. Primarily, the reason is the strong ability to secrete proteins, which allows to obtain >20 g of protein per liter of a medium (Harwood, Cranenburgh, 2008). Furthermore, various Bacillus species produce neutral and alkaline proteases (Anandharaj et al., 2016; Rehman et al., 2017), and this property is important for the industry. Proteases of Bacillus members have unique characteristics enabling their use in many industrial sectors. Consequently, proteases from various Bacillus species are responsible for ~60 % of all the sales of enzymes worldwide. Because of the wide ranges of pH and temperature corresponding to good activity and stability, these enzymes are used in the detergent industry (Porres et al., 2002). For this purpose, enzymes should be resistant to an alkaline medium and retain their activity in the presence of inhibitors, including oxidants and surfactants. In addition, the proteases isolated from the strains of Bacillus are suitable for the food industry for preparation of biologically active peptides and processing of various food products (Latiffi et al., 2013; Ke et al., 2018). Another feature of these proteases is stability in organic solvents and the consequent suitability for organic synthesis (Hu et al., 2013). Owing to the high commercial significance, a large number of patents deal with the strains of Bacillus (see the Table).

**Table 1.**
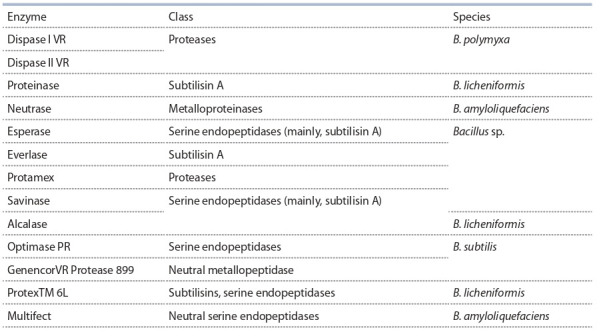
Industrial subtilases obtained from Bacillus species

The widespread manufacture of proteases by means of Bacillus strains is due to the economic efficiency of these strains. Additionally, it is possible to utilize the byproducts of agricultural production as a substrate for these strains, e. g. molasses of sugarcane and corn starch for submerged fermentation (Shikha et al., 2007) or various types of bran and solid residues for solid-state fermentation (Shivasharana, Naik, 2012).

## The search for alkaline serineproteases in nature

Proteases are commercially important proteins responsible for the lion’s share of protein manufacture. They have many applications, and each technological process has its specific features and requirements for the enzymes used. Besides, the unrelenting interest in these proteins is due to the search for enzymes that are not covered by patents, albeit with properties that are not necessarily better than those of the existing enzymes. Accordingly, a huge number of research papers on this topic is published every year. The largest number of genes of alkaline serine proteases has been discovered in the genomes of bacteria from the genus Bacillus. The second place in terms of isolation of proteases belongs to Actinomycetes. A substantial number of researchers also seek alkaline proteases of fungal origin (Sharma et al., 2017). In the latest articles, the emphasis is on the search for enzymes having a keratinase activity because of increased interest in the processing of keratin-containing residues, e.g., feathers.

The source of one of the promising genes encoding a serine protease is strain Bacillus licheniformis NMS-1 extracted from soil near a natural hot spring in Sri Lanka (Mathew, Gunathilaka, 2015). This protein serves for the creation of detergents. Closely related strain B. licheniformis K7A expressing an alkaline protease was obtained in another study (Hadjidj et al., 2018). Analysis of the expressed protein revealed that it has the highest activity at pH 10 and 70 °C. The enzymatic activity is higher than that of commercial preparations of Alcalase and Thermolysin. Another serine protease was found in the genome of the bacterium Bacillus amyloliquefaciens FSE-68 isolated from a starter culture for soy fermentation in South Korea. Its amino acid sequence was determined by liquid chromatography with electrospray ionization tandem mass spectrometry (LC/ESI-MS/MS) and by whole-genome sequencing. In comparison with a homolog, i. e., well-studied subtilisin BPN from B. amyloliquefaciens, that enzyme showed slightly higher stability in the absence of calcium ions (Cho, 2019). The protein isolated from alkaliphilic strain Bacillus luteus H11 manifested proteolytic activity at NaCl concentration up to 5 M, temperature 45 °C, and pH 10.5 (Kalwasińska et al., 2018). In China, during a screening of the bacteria obtained from industrial fermentation of soy, investigators isolated strain B. subtilis MX-6, which overexpresses a nattokinase-like protein (Gulmez et al., 2018). 

Numerous recent studies on the search for new versions of proteases of bacterial or fungal origin can be discussed ad infinitum. Therefore, for a variety of reasons, advances in the manufacture of proteolytic enzymes are still relevant today. This is especially true for developing countries, which strongly wish to increase the proportion of industrial products, including biotechnological ones, on their domestic markets. An especially large number of studies in this field has been published by research groups from India. At present, such studies are virtually absent in Russia.

## Genetic engineering of subtilisin

Subtilisin is the industrial enzyme that has probably been studied the most by both statistical and directed mutagenesis. The applications of subtilisin have expanded constantly since the start of its manufacture. To meet the needs of the industry, subtilisin characteristics had to be improved. In the early 1980s, the methods for directed engineering of proteins started to develop rapidly. As a result of application of these methods to subtilisin, mutations of more than a half of its 275 amino acid residues had been described before the year 2000 in scientific literature. Patents contain even more such accomplishments, and, undoubtedly, an even greater number of findings is buried in the freezers of biotech companies. Subtilisins represent a large class of microbial serine proteases, but the most mutagenized proteases are those from B. amyloliquefaciens (BPNP), B. subtilis (subtilisin E), and Bacillus lentus (Savinase).

Protein engineering involves several effective methods that include rational design and directed evolution. The former usually implies the methods of site-directed mutagenesis for replacing specific amino acid residues in a protein; this approach can help to obtain proteins with desired properties, such as higher thermal stability (Jaouadi et al., 2010; Huang et al., 2015). Besides, protein engineering can help to elucidate substrate recognition and point to possible applications of an enzyme (Jaouadi et al., 2014). On the other hand, directed evolution is based on the execution of sequential cycles of mutagenesis and selection (Liu et al., 2014). Thus, researchers may obtain enzymes with higher activity and stability under various conditions, including extreme pH and temperatures, nontraditional media, and modified specificity toward given substrates.

## Stability of subtilisin

Its stability has been urgently needed for its manufacture; in this regard, such studies have become widespread. An interesting feature of subtilisin is that its biosynthesis requires participation of its N-terminal pro-domain (Ikemura et al., 1987). Folding of mature subtilisin without the pro-domain is possible theoretically but will take thousands of years.

An important characteristic of subtilisin is its tremendous dependence on calcium (Voordouw et al., 1976; Genov et al., 1995). The universal characteristic of subtilisins is the presence of one or more sites for calcium binding. High-resolution X-ray structures of subtilisin BPNP and of several homologs (Bode et al., 1987; Betzel et al., 1992) have uncovered the details of a conserved calcium-binding site, which is called site А. Calcium in this site is coordinately bound by five carbonyl atoms of oxygen and an asparagine acid residue. Four of the oxygen atoms are provided by the loop containing amino acid residues 75–83. The geometry of the ligands is a pentagonal bipyramid, whose axis crosses the carbonyl groups of amino acid residues 75 and 79. On one side of the loop, bidentate carboxylate (D41) is located, and on the other, the N terminus of the protein and side chain Q2. Seven coordinate distances vary from 2.3 to 2.6 A, the shortest of which involves aspartyl carboxylate

The second ion-binding site (site B) is located 32 A away from site A in a shallow cleft between two segments of the polypeptide chain near the molecule’s surface. The coordination geometry of this region bears a striking resemblance to a distorted pentagonal bipyramid. Three of the formal ligands are derivatives of a protein and include an oxygen atom of carbonyl group E195 and two oxygen atoms from the carboxylate of the D197 side chain. Four water molecules complete the first coordination sphere.

Given that the dependence on calcium is undesirable, some research has been conducted to obtain stable subtilisins that do not depend on the presence or absence of calcium in solution. One research group (Strausberg et al., 2005) modified the amino acid sequence of subtilisin with a damaged calcium-binding site for increasing this enzyme’s stability. As a result, they obtained a mutant enzyme that is 15,000-fold more stable than the original protein. To this end, 12 mutations were introduced into the gene of this enzyme.

**The latest research on the modification of alkaline serine proteases**

In spite of substantial progress in the development of customized properties of alkaline serine proteases, the work on their modification continues to this day. For instance, in one study (Zhao, Feng, 2018), via directed evolution, the authors obtained seven mutants (P9S, A1G/K27Q, A38V, A116T, T162I, S182R, and T243S) of a protease extracted from Bacillus pumilus BA06. They all possessed a higher proteolytic activity toward casein and a synthetic peptide substrate at 15 °С. Except for T243S, thermal stability of these mutant enzymes did not decrease relative to the wild-type enzyme. Combinations of mutations further increased the specific caseinolytic activity. Double mutants P9S/K27Q and P9S/T162I showed approximately a fivefold increase in the caseinolytic activity at 15 °C almost without a loss of thermal stability (Zhao, Feng, 2018). In another study by the same group (Zhao et al., 2016), directed mutagenesis was performed on the alkaline protease of B. pumilus. The resultant double mutant (W106K/ V149I and W106K/M124L) possessed 2.5-fold higher activity in comparison with the original enzyme at 15 °C, whereas its stability at 60 and 70 °C was 2.7-fold and 5-fold higher, respectively (Zhao et al., 2016).

During a comparison of halotolerant subtilisins with unstable ones, researchers discovered six amino acid positions where polar amino acid residues were replaced with nonpolar ones. The researchers hypothesized that these substitutions may lead to higher thermal stability. To test this hypothesis, they carried out mutagenesis of the alcalase from strain B. subtilis No. 16 and subtilisin Carlsberg. As a result, there was respectively 1.2-fold and 1.8-fold greater resistance of the enzymes to higher salt concentrations (125 g/L) (Takenaka et al., 2018). In another work (Ashraf et al., 2019), a serine protease from Pseudomonas aeruginosa was modified at two positions (A29G and V336I); as a consequence, they achieved a 5 °C increase in the temperature of observed residual activity and 1.4-fold enhancement of the catalytic activity (Ashraf et al., 2019). In yet another study (Gong et al., 2017), statistical mutagenesis of an alkaline-protease gene discovered during a metagenomic analysis increased the enzymatic activity by 6.6-fold.

## Preparation of proteases in the strains of Bacillus spp.

The Bacillus bacteria have been the main microbial producers of serine proteases throughout the whole period of their practical use. Cultivation conditions and composition of the media play an important role in the production of enzymes by microbes (Abidi et al., 2008). To achieve high and commercially significant expression of proteases, it is crucial to find the conditions for growth and induction (Sharma et al., 2015). There is no universal medium suitable for all producer strains. Each microorganism or strain has unique specific conditions for maximal production of a given enzyme. Let us review various parameters of cultivation in more detail.

## Composition of media

Carbon and nitrogen are the main components of a medium and act as major stimulators of microbial growth and synthesis of enzymes. The most widespread source of carbon and often the cheapest (after starch) is glucose; however, during its consumption, the effect of catabolic repression of many biosynthetic processes may emerge in the cell. The highest production of the enzyme by bacterial strain AKS-4 is observed at a glucose concentration of 1 %. Under these conditions, the level of expression of the protease reaches 59.10 U/mL (Sharma et al., 2015). Higher production of proteases in Bacillus pseudofirmus AL-89 was observed after glucose addition, whereas for Nesterenkonia sp., the synthesis of protease AL-20 was found to be suppressed in the presence of glucose (Gessesse et al., 2003). 

The highest production of alkaline protease (2450 U/mL) in B. licheniformis was achieved in a medium containing 60 g/L glucose. A further increase in its concentration led to an insignificant decrease in the production of the enzyme. Glucose at a high concentration inhibited the synthesis of the enzyme in Streptomyces ssp., and concentration 0.5 % was optimal for the production of the enzyme, whereas 1 % was optimal for growth (Mehta et al., 2006). Production of a protease in P. aeruginosa MCMB-327 in a soy-tryptic medium decreased by 95 and 60 % after the addition of glucose and fructose, respectively (Zambare et al., 2011). In another work (Sharma et al., 2014), investigators tried various sources of carbon, such as glucose, lactose, galactose, and starch, for the production of a protease by Bacillus aryabhattai K3. The highest production of the protease (622.64 U/mL) was observed with lactose (10 g/L) as a carbon source (Sharma et al., 2014). Similarly, in yet another study (Dodia et al., 2006), researchers found that for most of the analyzed isolates, secretion of the enzyme is optimal with lactose as a carbon source. B. licheniformis BBRC 100053 also manifested higher productivity in terms of a protease in culture media containing lactose as a carbon source (Nejad et al., 2010).

Aside from simple sugars, investigators tried other carbon sources for the production of proteases. The addition of 5 % of starch resulted in the highest production of a protease by Bacillus sp. 2–5 (Khosravi-Darani et al., 2008). Strain Bacillus clausii No. 58 grew well on various carbon sources based on starch (Kumar et al., 2004). Corn starch at 0.5 % yielded the highest productivity in terms of the protease, followed by wheat flour and wheat bran. Nonetheless, the addition of potato starch lowered the titer of the protease, possibly because of the presence of protease inhibitors in potato (Kumar et al., 2004). Wheat flour as a sugar source gave good results on the production of proteases by Bacillus sp. (Chu, 2007). Bacillus laterosporus synthesizes proteases while utilizing various carbon sources; the best sources of carbon for the secretion of the protease are soluble starch, trisodium citrate, citric acid, and glycerol (Usharani, Muthuraj, 2010).

Nitrogen sources also significantly affect the yield of a desired protein, and optimal sources vary among different strains. The highest level of protease production by strain Bacillus cereus 146 was observed in the presence of a beef extract as a nitrogen source. The presence of a yeast extract, peptone, and tryptone improved the growth parameters of cultures, but the amount of the desired protein was still modest (Shafee et al., 2005). It was demonstrated in another study that tryptone increases the protease synthesis by strain Bacillus sp. (Srinivasan et al., 2009). Peptone was found to be optimal for the production of a protease by B. licheniformis BBRC 100053 (Nejad et al., 2010). The yeast extract causes the biggest increase in the production of enzymes by Bacillus sp. (Prakasham et al., 2006). In case of Bacillus sp. APP1, among all the tested sources of organic nitrogen, soy protein meal noticeably raised the synthesis of an extracellular protease (Chu, 2007). Some authors (Jaswal et al., 2008) also reported that the addition of soy protein meal gave the best results in comparison with casein, gelatin, and peptone for the expression of a protease by Bacillus circulans. When casein, peptone, the yeast extract, and a beef extract were tested as a nitrogen source for the synthesis of a protease by bacterial strain AKS-4, the highest expression was observed in the presence of casein. Among the various sources of organic nitrogen, nonfat milk gave the highest yield of a protease in the case of Bacillus caseinilyticus, followed by a malt extract, peptone, and the yeast extract. Ammonium chloride as an inorganic source of nitrogen inhibits the synthesis of a proteinase (Mothe, 2016)

## The influence of pH and temperature on the expression levels of proteases

The impact of pH on the expression level of a desired product is unique for each producer strain. For example, for the expression of proteases in Bacillus sp. MIG (Gouda, 2006) and B. cereus SIU1(Singh et al., 2010), weakly acidic pH (6.3–6.5) was found to be optimal. In a weakly alkaline medium (pH 8.0–8.5), researchers noted the highest levels of expression for B. licheniformis IKBC-17 (Olajuyigbe et al., 2005), B. subtilis IKBS 10 (Olajuyigbe et al., 2005), Bacillus macerans IKBM-11 (Olajuyigbe et al., 2005), and B. amovivorus (Sharmin et al., 2005). In one study on eight isolates of Bacillus (Dodia et al., 2006), it was revealed that the best conditions for the growth of bacteria involve pH 9.0, whereas the optimal pH value for the secretion of the enzyme varies between 8.0 and 10.0. pH 9 was found to be optimal for the production of proteases in Bacillus sp. (Prakasham et al., 2006), Bacillus sp. APP1 (Chu, 2007), and B. proteolyticus CFR3001 (Bhaskar et al., 2007). Higher starting pH was set up for the production of a protease by B. licheniformis TISTR 1010 (pH 10.0) (Vaithanomsat et al., 2008), for B. circulans (pH 10.5) (Jaswal et al., 2008), and for Bacillus sp. 2–5 (pH 10.7) (Khosravi-Darani et al., 2008).

Temperature is also a crucial parameter, and the optimal temperature is unique for each strain. For P. aeruginosa PseA (Gupta, Khare, 2007), B. licheniformis (Asokan, Jayanthi, 2010), Bacillus coagulans (Asokan, Jayanthi, 2010), B. cereus (Kebabcı, Cihangir, 2010), P. aeruginosa MCMB-327 (Zambare et al., 2011), P. chrysogenum IHH5 (Ikram-Ul-Haq et al., 2006), and A. oryzae 637 (Srinubabu et al., 2007), the optimal temperature for the synthesis of proteases is 30 °С. A lower optimal temperature (25 °С) characterizes B. circulans (Jaswal et al., 2008) and Microbacterium sp. (Thys et al., 2006), whereas in B. cinerea, the highest expression was documented at 28 °С (Abidi et al., 2008). At 37 °С, the maximal level of expression was observed for the strains of Bacillus amovivorus (Sharmin et al., 2005), B. proteolyticus CFR3001 (Bhaskar et al., 2007), Bacillus aquimaris VITP4 (Shivanand, Jayaraman, 2009), and B. subtilis Rand (Abusham et al., 2009); at 40 °С for Bacillus sp. 2–5 (Khosravi-Darani et al., 2008), Vibrio pantothenticus (Gupta et al., 2008), and Streptomyces roseiscleroticus (Shivanand, Jayaraman, 2009); and at 50 °С for Bacillus sp. APP1 (Porres et al., 2002) and B. subtilis BS1 (Shaheen et al., 2008).

## Expression of alkaline serine proteases in yeast

The synthesis of proteases is possible not only in the strains of Bacillus but also in other bacteria and in yeast, e. g., in the strains of Pichia pastoris. These strains naturally do not have a specific activity; for this reason, they require modification by genetic engineering. There are few such studies, and for the most part, they are aimed at obtaining fungal proteases or medically important proteases.

In one study, B. Liu et al. (2014) performed an analysis of expression of the keratinase gene in B. licheniformis BBE11-1 in three heterologous expression systems: in Escherichia coli, B. subtilis, and P. pastoris. The highest (best) level of expression was seen in B. subtilis (3010 U/mL); this level was threefold higher than that in P. pastoris. It should be noted that the cultivation of B. subtilis does not involve methanol, and cultivation duration is twofold shorter. In another study (Radha, Gunasekaran, 2009), there is a description of comparative cloning of the keratinase from B. licheniformis MKU3 in B. megaterium and P. pastoris. As a result, those authors obtained comparable activities of the final culture with the concentration of the desired protein at ~0.35 g/L. The protein from P. pastoris was subject to glycosylation. It should be mentioned that cultivation in a bioreactor was not described in that work. Similar results were published about the expression of the keratinase from B. licheniformis PWD-1 (Cheng et al., 1995).

In one work (Lin et al., 2009), researchers investigated the expression of the keratinase from P. aeruginosa in P. pastoris. The expression level was approximately 0.5 g of the protein per liter. In this case, the protein did not undergo glycosylation. In another work (Zhou et al., 2017), protein subtilisin QK (from B. subtilis QK02), which is highly similar to nattokinase, was cloned in P. pastoris GS115. Their objective was to obtain a protein with thrombolytic effects. As a result, they achieved a high concentration of total protein in the final supernatant (7.6 g/L). In this work, pH was maintained at 5.0, whereas in other studies (Liu et al., 2014) and (Porres et al., 2002) – the absence of pH control caused a pH increase, resulting in inhibition of microbial growth and a drop in keratinase concentration in solution. A similar picture was observed in a study by H.H. Lin et al. (2009).

Cloning of the alkaline protease from thermophilic bacterium B. stearothermophilus F1 was also conducted in P. pastoris GS115 (Latiffi et al., 2013). The resultant activity was 4.13 U/mL; judging by the obtained molecular weight, the protein was not glycosylated. In one study (Ke et al., 2018), the gene of the alkaline protease from fungus Aspergillus sojae was expressed in P. pastoris, and the final activity reached 400 U/mL.

The level of expression of a desired protein is strongly affected by codon usage too. In one work (Hu et al., 2013), as a result of optimization of codon usage in a gene, the level of expression of the desired protein was raised relative to the original gene. That study, however, does not present the data on the cultivation under controlled conditions of a bioreactor. An increased copy number of the expression cassette also allows for improving the yield of a desired protein, as exemplified by a serine protease from the fungus Trichoderma koningii (Shu et al., 2016).

In conclusion of this section, it is worth noting that the highest accumulation of alkaline serine proteases is greater in the P. pastoris expression system than in the E. coli expression system, but lower than that in standard B. subtilis strains. At the same time, the industrial strains of Bacillus spp. out perform both P. pastoris expression systems and B. subtilis by more than an order of magnitude. A 2005 patent (Shih, 2005) describes strain B. licheniformis T1, which ensures the expression level of a protein at 16 g/L, whereas the highest concentration of keratinase produced in P. pastoris is approximately 0.1–0.2 g of the desired protein per liter.

## Conclusion

Alkaline serine proteases of the subtilisin family are widely applied in various industrial sectors. Proteases isolated from Bacillus bacteria constitute approximately 60 % of all enzyme sales across the globe.

Currently, native enzymes, i. e., those found in nature, are hardly used and have been ousted by the proteins modified via genetic engineering and thus possessing better properties, e. g., thermal stability, general resistance to detergents and specific resistance to various oxidants, high activity in various temperature ranges, independence from metal ions, and stability in the absence of calcium.

At present, diverse strains of Bacillus serve as microbial producers of alkaline serine proteases. Most of them originally had the desired activity, which has been enhanced by mutagenesis or genetic engineering. Among the producer strains, the species having the GRAS (generally regarded as safe) status dominate, that is, those that are even considered safe to eat: mostly B. subtilis and B. licheniformis. The strains that originally did not possess a protease activity still cannot be brought to the level of the native producers, even by means of genetic engineering technologies.

In literature, there are reports of the efforts to construct microbial producers of alkaline serine proteases on the basis of a methylotrophic strain of P. pastoris. In comparison with the expression of the same genes in the genetically engineered strains of B. subtilis, the results have turned out to be noticeably worse. From the above observations, it can be concluded that for constructing the strains effectively producing desired alkaline proteases, it is necessary to employ Bacillus-based expression systems. These strains need optimization of the properties of the expressed enzyme and of its expression level by methods of directed and statistical mutagenesis. Patentable microbial producers of an alkaline serine protease (subtilisin A) can be created via a search for new natural strains or may be constructed from the strains that lost patent protection.

## Conflict of interest

The authors declare no conflict of interest.
